# A database of whole-body action videos for the study of action, emotion, and untrustworthiness

**DOI:** 10.3758/s13428-013-0439-6

**Published:** 2014-03-01

**Authors:** Bruce D. Keefe, Matthias Villing, Chris Racey, Samantha L. Strong, Joanna Wincenciak, Nick E. Barraclough

**Affiliations:** 1 Department of Psychology, University of York, Heslington, York YO10 5DD UK; 2 Department of Linguistics, University of Tübingen, Tübingen, Germany; 3 School of Optometry and Vision Science, University of Bradford, Bradford, West Yorkshire BD7 1DP UK; 4 Department of Psychology, University of Hull, Cottingham Road, Hull, HU6 7RX UK

**Keywords:** Action, Emotion, Untrustworthiness, Database, Gender, High-definition, Photo-realistic, 3D, HD

## Abstract

We present a database of high-definition (HD) videos for the study of traits inferred from whole-body actions. Twenty-nine actors (19 female) were filmed performing different actions—walking, picking up a box, putting down a box, jumping, sitting down, and standing and acting—while conveying different traits, including four emotions (anger, fear, happiness, sadness), untrustworthiness, and neutral, where no specific trait was conveyed. For the actions conveying the four emotions and untrustworthiness, the actions were filmed multiple times, with the actor conveying the traits with different levels of intensity. In total, we made 2,783 action videos (in both two-dimensional and three-dimensional format), each lasting 7 s with a frame rate of 50 fps. All videos were filmed in a green-screen studio in order to isolate the action information from all contextual detail and to provide a flexible stimulus set for future use. In order to validate the traits conveyed by each action, we asked participants to rate each of the actions corresponding to the trait that the actor portrayed in the two-dimensional videos. To provide a useful database of stimuli of multiple actions conveying multiple traits, each video name contains information on the gender of the actor, the action executed, the trait conveyed, and the rating of its perceived intensity. All videos can be downloaded free at the following address: http://www-users.york.ac.uk/~neb506/databases.html. We discuss potential uses for the database in the analysis of the perception of whole-body actions.

**Electronic supplementary material:**

The online version of this article (doi:10.3758/s13428-013-0439-6) contains supplementary material, which is available to authorized users.

Successful social interaction requires that we accurately determine both the emotions and internal mental states of other individuals. Both body movements and facial expressions can each provide information on the underlying emotional state of the agent (Ekman, 1965; Ekman & Friesen, 1975), although the majority of research has focused upon the perception of facial expressions. Characteristic bodily movements, however, can signal an intention that cannot be derived from facial expressions (e.g., approach and avoidance behavior). Furthermore, the ability to derive information from bodily actions can be used in critical situations where the face is occluded (e.g., when the face is turned away, the actor is wearing a mask or hat, etc.) or the individual is too far away to reliably detect face movements or face shape.

Several recent studies have started to examine the mechanisms underlying the perception of emotions conveyed by body actions (reviewed in de Gelder, 2006; de Gelder et al., 2010). While databases exist for the study of emotion from point-light walkers (where information about action is available only from the motion of points of light typically placed on major joints; e.g., Atkinson, Dittrich, Gemmell, & Young, 2004; Clarke, Bradshaw, Field, Hampson, & Rose, 2005; Ma, Paterson, & Pollick, 2006), few databases exist for the extensive study of emotions and other traits derived from naturalistic whole-body actions (Atkinson et al., 2004; O’Toole et al., 2005). Atkinson et al.’s database of emotional whole-body actions contains both point-light and full-light stimuli; however, this database is limited to 150 full-light stimuli, and the 10 actors execute only one un-defined action in order to convey different emotions. Notably, Atkinson et al.’s stimuli appear unnaturalistic, as actors were required to wear special suits and masks in order to facilitate the conjoint recording of both point-light and full-light versions of each of the actions. While full-light, naturalistic, video databases do exist, they have tended not to include emotional actions (O’Toole et al., 2005; Umla-Runge, Zimmer, Fu, & Wang, 2012). O’Toole et al.’s database included a large number of actors performing two actions (284 videos); these actions, however, did not convey any specific emotions. Further, Umla-Runge et al.’s database includes a large number (784) of goal-directed actions executed by the hand and arm; however, they also do not convey specific emotions. Other techniques have combined motion capture and three-dimensional (3-D) animation software to provide computer-generated full-body actions (e.g., Dekeyser, Verfaillie, & Vanrie, 2002; Vanrie & Verfaillie, 2004). These techniques provide precise control (e.g., viewpoint manipulation) over computer-generated avatars but do not provide the same degree of realism that is afforded by full-light recordings of human actions, such as those provided in the database presented here.

In order to address this shortage in available high-definition, photorealistic, but flexible databases of emotional human actions and provide useful stimuli for a range of different investigations into action perception, we generated an action database and present the details here. Twenty-nine actors (19 female) performing six different actions (walking, standing and acting, picking up a box, putting down a box, jumping, and sitting down) while conveying different traits (anger, fear, happiness, sadness, untrustworthiness, and neutral) were filmed in high-definition (HD) while the face was not visible to the camera. The practicality of generating large numbers of high-quality action videos restricted the number of different actions that could be filmed; however, we wanted to ensure the greatest flexibility for future empirical studies of action perception. We filmed six different actions, thereby ensuring that different types of actions were represented in the database. We included actions executed on the spot with no substantial translatory movement (e.g., sitting), so that, in theory, several of these actions could be superimposed within one scene, each with a different position on the *x*-axis such that no actions would overlap; we also included an action translating across the field of view (walking), as much earlier research in action recognition has focused on this action type. We also filmed both transitive actions (picking up a box) and nontransitive actions (e.g., standing and acting) for inclusion in the database, as it is known that that the presence of an object can influence action representation (Perrett et al., 1989; Umiltà et al., 2001). We included both picking up box actions and putting down box actions, so that the database contained actions with similar (albeit opposite) kinematics with different goals; such stimuli have been useful in the development of computational models of action recognition (Barraclough, Keith, Xiao, Oram, & Perrett, 2009; Fleischer, Caggiano, Thier, & Giese, 2013). Emotions were chosen to ensure that both positive and negative valence emotions were represented. Furthermore, recent studies of face evaluation (e.g., Oosterhof & Todorov, 2008) suggest that perception of emotion and untrustworthiness are interlinked. We, therefore, included in the database actions executed in an untrustworthy fashion in order to provide stimuli for future evaluation of the interactions between emotion and untrustworthiness conveyed by whole-body actions.

Filming using a 3-D camera was performed in a green-screen studio in order to isolate the action information from all other contextual and environmental detail during postprocessing. To provide independent information about the degree of the trait conveyed in each video, we obtained trait intensity ratings for all the actions (2,783 in total), presented in two dimensions (2-D), from 10 observers; these independent ratings have been used to name each video and allow stimuli to be selected on the basis of their average perceived intensity. Furthermore, in order to confirm that the traits conveyed by the actions could be identified, we asked a second set of 10 independent observers to identify the trait conveyed by each action. We believe that this library will be useful for the study of emotions, trustworthiness, gender, and identity derived from whole-body actions. The use of HD video allows flexible use of the stimuli, as well as ensuring that single frames taken from the video are of high enough quality to be presented as stills. Thus, the database can be used to investigate the contribution of static and dynamic body postures to the perception of body actions. The database, supplementary supporting material and all data pertaining to stimulus rating and identification may be downloaded from http://www-users.york.ac.uk/~neb506/databases.html.

## Method

### Video preparation

#### Actors

Twenty-nine actors (19 female, 18–38 years of age, mean age = 22.4, *SD* = 5.0) were filmed performing actions while conveying different traits. Twelve of the actors were studying drama at the University of York; the remaining 17 actors were other staff and students at the University of York. All actors wore a gray t-shirt and dark trousers and shoes. Actors gave informed consent to allow the distribution of their videos publically and were paid for their time.

#### Filming

Actors were filmed in an evenly lit green-screen studio. Actions were performed with the profile of the actor at 45° to a custom-built 3-D camera in order to exclude facial information. The 3-D camera was composed of two Panasonic TM900 digital cameras (each filming at 1,920 × 1,080 pixels, and 50 fps progressive scan, equivalent to full HD) mounted on a solid aluminium plate and set to a 66-mm parallel interaxial distance (IAD). The height of the camera was set to 1.22 m, and it was placed 5.1 m from the actor.

#### Acting

**Fig. 1:**
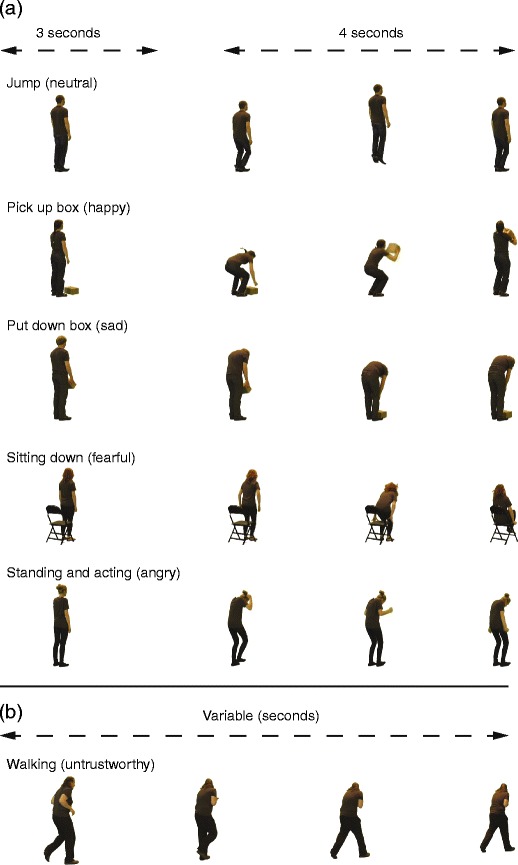
An illustration of sample frames from example actions and traits. **a** Actions that were performed in one position in space. These actions consisted of 3 s of inactivity followed by 4 s of action execution. **b** Walking actions where the actor moved across space. The walking action varied in duration (*M* = 3.10 s, *SD* = 0.88 s, range = 0.94– 6.52 s)

Traits conveyed included four emotions (anger, fear, happiness, sadness), untrustworthiness, and neutral (no specific trait), examples of which are illustrated in Fig. 1. For all traits except neutral, three levels of increasing trait intensity were recorded (low, medium, and high). The combination of traits and trait intensity produced, in total, 16 trait categories: 5 traits (anger, fear, happiness, sadness, untrustworthiness) x 3 trait intensities (low, medium, high), and the 1 neutral trait. For each trait and level of trait intensity, actors performed six different actions: walking, standing and acting, picking up a box, putting down a box, jumping, and sitting down (see Fig. 1). For all actions, actors were asked to convey the trait in whatever manner they wished. During the “standing and acting” action, they were instructed not to move from their position. The number of actions generated from this procedure included 29 actors × 16 trait combinations [trait × trait intensity] × 6 actions—in total, 2,783 actions (one video had to be removed due to a filming error).

Autobiographical recall, a mood induction procedure (MIP), was used to help actors convey the appropriate trait during filming; this technique has been shown to be effective in eliciting affective states (Jallais & Gilet, 2010; see Westermann, Spies, Stahl, & Hesse, 1996, for a review). Using these procedures, actors recalled and wrote down past events that had elicited a particular trait at each level of trait intensity (low, medium, high). The actor then held the relevant event in mind while acting. The written evidence of the MIPs was only ever seen by the participating actor and was taken with them after filming. No MIP procedure was used for the neutral category, in which actors were simply asked to perform the actions in a neutral fashion. Filming was blocked by traits, which were acted in the following order: neutral, fear, sadness, anger, untrustworthiness, and happiness. In each block, the actors performed the actions in order from lowest to highest trait intensity. Blocks were separated by short breaks in order to allow for the MIP.

Prior to performing each action, actors were asked to stand still with their hands by their side. Acting start and finish times were 4 s apart and were indicated to the actor by an audible “beep.” Enough filming time was allowed so that all actions were preceded by 3 s of actor inactivity. The only exception to this procedure was for the walking actions. Here, the actor started to walk into the camera field of view when a “beep” was heard; the action ended when the actor had reached the other side of the room (outside the field of view of the camera).

#### Editing

Sony Vegas Pro 10 (Sony Creative Software Inc., Middleton, WI) was used to edit the video footage. Chroma key compositing was used to remove the green background and isolate the actor from all other contextual information. Each video was edited to 7-s duration, comprising an initial 3 s where the actor stood still, and a subsequent 4 s from the onset of the action (see Fig. 1). The nature of the different actions (except walking) meant that some actions would typically last for the entire 4 s (e.g., standing and acting), whereas others could be completed before the end of the 4-s window (e.g., sitting down). For the walking stimuli, the video started on the first frame when the actor’s whole body was visible from the camera and finished a frame after the actor walked out of shot. The length of the walking video was therefore variable and determined by the speed at which the actor walked (*M* = 3.10 s, *SD* = 0.88 s, range = 0.94–6.52 s).

### Intensity ratings experiment

#### Participants

Participants were University of York students and staff (5 females, 5 males; 19–27 years of age, *M* = 23.3, *SD* = 2.8; 3 were also actors for the stimuli). We chose a sample size of 10 on the basis of a power analysis using previously reported effect sizes from similar work (Atkinson et al., 2004; *η*_p_^2^ = .327). All participants had normal or corrected-to-normal vision, gave informed consent, and were paid for their participation. The experiment was approved by the ethics committee of the Department of Psychology, University of York, and was performed in accordance with the ethical standards laid down in the 1990 Declaration of Helsinki.

#### Stimuli

All stimuli were prepared in 2-D by using the video information from only the left Panasonic TM900 camera. Stimuli were generated by editing the 7-s videos to 2 s. Each 2-s video started at the onset of the action, 3 s into the original 7-s video. For walking actions, the 2-s video started on the first full frame with the actor in view. These short 2-s videos were used to ensure that the overall duration of the rating experiment was kept to manageable proportions. All videos files were compressed with WMV 9 advanced profile compression: 1,920 × 1,080 pixels, 50 fps, progressive scan, variable bit rate, 8.4 Mbps, and 72 % quality. A PC running MATLAB 2010a (The MathWorks, Natick, MA) and Psychtoolbox (Brainard, 1997; Kleiner, Brainard & Pelli 2007; Pelli, 1997) was used to control the experiment, display the stimuli, and record participants’ responses. A custom movie playback engine was used alongside Psychtoolbox to play the videos.

#### Experimental procedure

Participants sat in a dimly lit room approximately 57 cm away from a 24-in. TFT monitor (Acer GD245HQ, 1,920 × 1,080 pixels, 100-Hz refresh rate) on which all visual stimuli were presented. The experiment consisted of five blocks of testing that lasted approximately 1h each and were completed on separate days. Blocks were separated into 5- to 10-min subblocks, in order to provide the participant with rest breaks. During each block of testing, videos of actors conveying only one trait (anger, fear, happiness, sadness, or untrustworthiness) were shown along with the videos of the actors performing the neutral actions. Neutral actions were included in every experimental block to examine participants’ ratings of anger, fear, happiness, sadness, and untrustworthiness for these actions. Neutral actions would be expected to be perceived with lower trait intensity than the other actions where the actor was asked to explicitly convey a trait. Each block therefore contained 696 videos (one block contained 695 videos), comprising all 29 actors, performing all six actions, at each of the three levels of trait intensity (low,medium, and high), and the neutral category. The order in which the stimuli were displayed within each block was randomized. Block order (trait) was counterbalanced across participants.

At the start of each trial, the video was displayed on screen (subtending a maximum of 18.9° × 5.0° of visual angle for an actor standing with hands by his or her side) for 2s, after which the screen turned black. The participant was instructed to rate the intensity of the trait conveyed by the actor on a 1–9 Likert scale (e.g., 1 = *not at all happy*, 9 = *extremely happy*, for the happy blocks of testing) and to indicate the response as quickly and accurately as possible on the keyboard number pad during a 2-s intertrial interval. It is important to note that this response range represents two ends of a continuum; one end of this continuum indicates that the particular trait being rated was present (e.g., 9), and the other that it was completely absent (e.g., 1). If no response was recorded during the intertrial period, the trial was marked as uncompleted and incorporated at a random point later in the testing block. The rating scale was always specific to the trait being judged, and participants were informed of this scale at the start of each block.

### Trait identification experiment

#### Participants

Participants were University of York students and staff (6 females, 4 males; 21–53 years of age, mean age = 27.1, *SD* = 9.6); no participants had taken part in the intensity rating experiment, and the number of participants was based upon a power analysis of the previous experiment. All participants had normal or corrected-to-normal vision, gave informed consent, and were paid for their participation. The experiment was approved by the ethics committee of the Department of Psychology, University of York and was performed in accordance with the ethical standards laid down in the 1990 Declaration of Helsinki.

#### Stimuli and procedure

Stimuli were the same 2,783 two-s duration action videos as those used in the intensity ratings experiment. Participants took part in a six-alternative forced choice experiment where they were required to classify each action video as one of the six traits. On each trial, a video was presented for 2 s; during a following period of 2 s, the participant was required to indicate which of six traits best represented the action by pressing a labeled key (anger, fear, happiness, neutral, sadness, or untrustworthiness). If no response was made during this time, the trial was incorporated at a random point later during testing.

All 2,783 videos were presented in a random order over 20 blocks of testing. Each block was just under 10min in duration, and breaks were given between blocks to reduce fatigue and help participants maintain attention on the task. Participants were instructed to complete only as many blocks as they felt comfortable with within a session (typically, a participant would complete 2–5 blocks during a day).

## Results

**Fig. 2:**
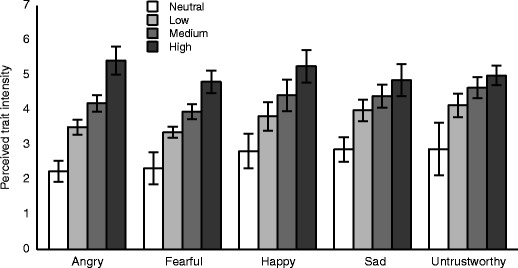
Plot showing interaction between trait and stimulus trait intensity. Error bars display ±95 % confidence intervals (Morey, 2008). Cases where the confidence intervals on one bar do not overlap with the mean of a different bar imply a significant difference in a pairwise *t*-test

**Fig. 3:**
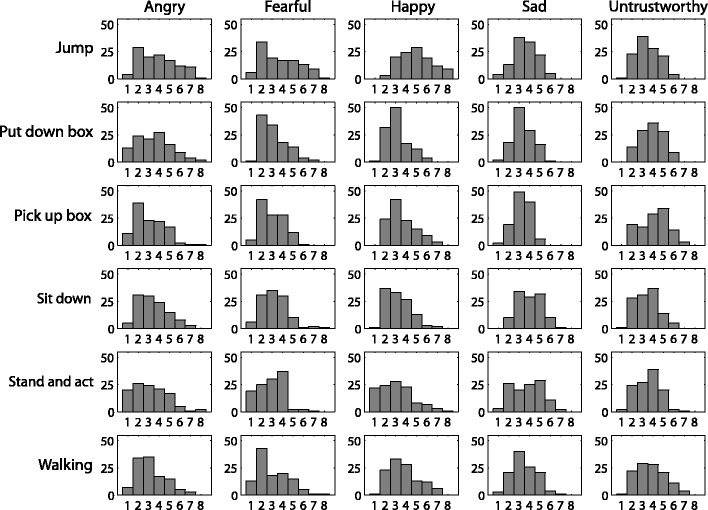
Histograms of videos in the database conveying different trait intensities. Neutral videos rated along the respective trait are included within each bar graph. The legend on the *x*-axis indicates conveyed trait, and the legend on the *y*-axis indicates action. Each subplot illustrates the availability within the database of actions conveying traits with different intensities. These data are available in table format within the database (number of stimuli by trait, action, and rating.xlsx). Videos of each action and trait were binned according to their mean perceived trait intensity (1 to <2, 2 to <3, 3 to <4, 4 to <5, 5 to <6, 6 to <7, 7 to <8, 8 to ≤9); bins are labeled along the subplot *x*-axes; the subplot *y*-axes indicate frequency of videos of that type

In order to examine whether instructions to the actors to convey different intensities of traits resulted in a range of action videos that actually conveyed different levels of trait, rating data was entered into a two-way ANOVA with the factors of trait (5) and stimulus trait intensity (4).^1^ Different traits were conveyed with similar intensities [main effect of trait: *F*(4, 36) = 2.52, *p* = .058, *η*_p_^2^ = .29]. Importantly, stimulus trait intensities were significantly different [main effect of stimulus trait intensity: *F*(1.1, 9.8) = 85.51, *p* < .0001, *η*_p_^2^ = .91; Greenhouse–Geisser correction applied]. This demonstrates that the actors were able to manipulate the intensity of the trait they conveyed according to the instructions they received and suggests that autobiographical recall might be useful in eliciting different affective states. Finally, participants’ rating of trait intensity was significantly different for different traits [trait × stimulus trait intensity interaction: *F*(12, 108) = 10.10, *p* < .001, *η*_p_^2^ = .53]. The range of trait intensity was largest for angry actions and smallest for untrustworthy actions, suggesting that participants found it relatively harder to distinguish high- from low-intensity untrustworthiness (or actors found it relatively harder to convey a range of different intensities of untrustworthiness; see Fig. 2). Despite these differences in the mean ratings of the different traits conveyed in the action videos, the database contains many videos of actions conveying several traits over a range of different intensities. The availability of these videos within the database are illustrated in Fig. 3, indicating the number of videos of each action, trait, and intensity combination (these data are available in table format within the database: number of stimuli by trait, action, and rating.xlsx). Removing from the analysis the 3 participants who were also actors did not change the statistical pattern of results observed.

### Trait identification data

**Fig. 4:**
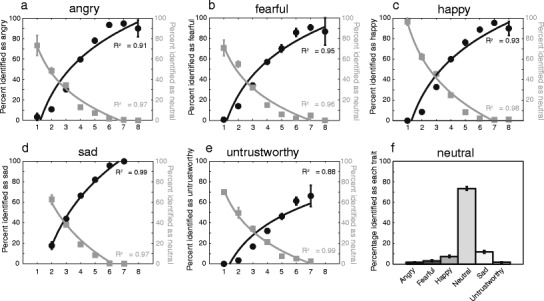
Identification accuracy of different traits. **a–e** Angry, fearful, happy, sad, and untrustworthy action videos were binned according to their mean perceived trait intensity (1 to <2, 2 to <3, 3 to <4, 4 to <5, 5 to <6, 6 to <7, 7 to <8, 8 to ≤9); bins are labeled along the *x*-axes. The percentage of the actions, within each bin, identified as the appropriate trait are illustrated on the *y*-axes (black circles). The percentage of the actions, within each bin, identified as neutral are also illustrated on the *y*-axes (gray squares). Error bars indicate the *SEM*, and functions are logarithmic curves. **f** Bar graph illustrating the percentage of the neutral stimuli identified as neutral or one of the five traits

In order to ensure that the action videos conveyed the traits that the actors intended (rather than opposite or different traits), we asked participants to identify the trait conveyed in each of the videos. As expected, the accuracy of trait identification quickly increased with the intensity of the conveyed trait (see Fig. 4a–e). Action videos conveying strong intensity traits (e.g., rated 7–9) were accurately identified (accuracy: anger 92 %, fear 89 %, happiness 93 %, sadness 98 %, untrustworthiness 64 %). When actions conveyed only weak trait intensity, they were less likely to be identified as the appropriate trait and more likely to be identified as neutral (Fig. 4a–e, gray lines). Neutral videos were identified as neutral in most cases (74 % of all neutral stimuli) and only occasionally misidentified, for example, as sad (12 %) or happy (7 %; see Fig. 4f).

For each of the five sets of stimuli conveying the five traits, accuracy of identification was tested with separate ANOVAs (trait × intensity bin). Identification of traits was significantly different for all stimulus sets (main effects of trait: all *F*s > 23.5, *p*s < .001, *η*_p_^2^ > .72); planned comparisons indicated that the appropriate trait was selected significantly (*p* < .05) more than all other traits in all cases apart from the untrustworthy actions, where they were not selected significantly more than the fearful actions, *F*(1, 9) = 4.45, *p* = .064, *η*_p_^2^ = .33, or the neutral actions, *F*(1, 9) = 0.89, *p* = .369, *η*_p_^2^ = .09. Untrustworthy stimuli, in some cases, can be misidentified with either fearful or neutral stimuli. Importantly, however, interactions between trait and intensity bin were significant for all stimulus sets, all *F*s > 19.33, *p*s < .001, *η*_p_^2^ > .68, reflecting significant increases in identification accuracy of the appropriate trait with intensity bin (simple main effects: all *F*s > 83.50, *p*s < .001, *η*_p_^2^ > .90). Neutral stimuli were significantly more often identified as neutral [ANOVA: *F*(1.8, 16.6) = 353.57, *p* < .0001, *η*_p_^2^ = .98; planned comparisons between neutral and all other traits: all *F*s > 315.53, *p*s < .0001, *η*_p_^2^ > .97]. Tables detailing the results of all statistical tests are included within the database (trait identification stats summary.docx).

### Availability of stimuli

All 2-D stimuli can be downloaded free from http://www-users.york.ac.uk/~neb506/databases.html. Three-dimensional format versions of these stimuli are available on request from the corresponding author. Further information provided at this site includes the following: (1) information on how stimuli can be downloaded and interpreted, and an explanation of all supplementary files within the database (readme.pdf), (2) information on each actor’s age, gender, and acting experience (actor details.pdf), (3) full frontal photographs of the face of each actor conveying a neutral expression (plus a restricted subset of actor faces conveying emotions), (4) raw and summary data on ratings of all videos from all observers (video rating data.xlsx, video identification data.xlsx), and (5) ancillary notes on stimuli (video error notes.xlsx, video names readme.pdf, walking video durations.xlsx).

When selecting stimuli, it is important to consider the response range used in the ratings task. The response range is diagnostic of whether particular traits are conveyed by the actions because it represents two ends of a continuum. One end of this continuum indicates that the trait was present (9), and the other that it was entirely absent (1). Rating data and trait identification data on every action video can be cross-referenced using the tables within the database (video identification data.xlsx).

## Discussion

We have described the generation, and assessment made by independent observers, of 2,783 videos of whole-body actions filmed in HD. We believe that these stimuli, which we provide at http://www-users.york.ac.uk/~neb506/databases.html, will be of use for theoretically motivated investigations of the processes underlying the perception of emotion, trustworthiness, identity, or gender from whole-body actions.

We conducted an experiment where observers rated the intensity of traits conveyed during all action videos. This experiment indicated that actors could convey traits with different intensities (e.g., from not at all untrustworthy to very untrustworthy) when instructed. Analyses of these data revealed that the intensity of the trait conveyed varied also with the trait type and the instructions to the actor (stimulus trait intensity). These differences and interactions, described in the Results section, reflect variances in the acting process. By conducting the rating experiment, however, we obtained an objective assessment of the intensity of the trait conveyed in every action video. As such, this assessment, along with the large number of actions filmed, enables the database to provide a range of actions varying on a continuum of conveyed trait intensity. It will be possible for researchers to select specific videos that convey specific levels of particular traits. Furthermore, this database allows multiple stimuli to be chosen on the basis of the gender of the actor, the action executed, and the trait conveyed.

We conducted a second experiment to ensure that the traits could be accurately identified. As was expected, the higher the intensity of the trait conveyed (i.e., a stronger signal), the more easy it was to identify the trait conveyed by the actor (cf. Atkinson et al., 2004). This monotonic increase was well modelled by a logarithmic function, indicating that participants were quickly able to identify traits when the strength of the trait signal increased. The untrustworthy actions were the least accurately identified, perhaps reflecting the difficulty for actors to portray this particular trait; several of the actors indicated that indeed this was the case. For low intensities, the trait was less accurately identified, due to there being less signal to detect. With lower intensity traits, participants were increasingly likely to indicate that there was no trait present (neutral). In addition, the “neutral” actions were typically identified as neutral, rather than portraying another trait. A few “neutral” actions were identified as sad; perhaps, as neutral actions convey no particular affect, and therefore show no particular characteristic movements, they can be occasionally misidentified as sad, an emotion characterized by reduced movements (Roether, Omlor, Christensen, & Giese, 2009).

Our stimuli were only 2 s long, and participants were given 2 s to respond (to keep the experiment to a manageable duration). In addition, participants were required to identify 2,783 videos, a particularly demanding task that took 20 blocks of testing over multiple days. Despite these demands, the degree of accuracy in trait identification we observed was high and is in line with the accuracy for the identification of other, equivalent stimuli (e.g., Atkinson et al., 2004). Atkinson et al. presented full-light whole-body actions conveying traits for longer (4.2–8s, *M* = 5.8 s), and participants were only required to identify 150 actions; they found that participants were able to identify angry actions with 86 % accuracy, fearful actions with 91 % accuracy, happy actions with 87 % accuracy, and sad actions with 87 % accuracy.

We believe this database represents an important new tool for theoretically motivated studies of the perception of whole-body actions. There are currently many available face databases and a few databases for studying actions using point-light stimuli (e.g., Ma et al., 2006). This database, however, is well suited to contribute to recent efforts directed to understanding the mechanisms underlying the perception of emotions and traits conveyed by bodies (for reviews, see, e.g., de Gelder, 2006; de Gelder et al., 2010).

It may be possible to use the stimuli from this database to study the principle dimensions underlying the perception of traits from whole-body actions. Currently, ratings of the actions in the database are only of the trait that the actor was conveying (with the exception of all the “neutral” actions). This method was adopted to gain information about the intensity at which the appropriate trait was conveyed by the actor. In face perception research, however, dimensional analyses are being employed to determine the relationship between the perception of facial emotions and facial traits. Analyses on faces assessed on multiple dimensions have shown that faces are evaluated principally on the dimensions of valence (trustworthiness) and dominance (Oosterhof & Todorov, 2008), as well as youthful attractiveness (Sutherland et al., 2013). In contrast, we know very little about how the perception of emotion and traits conveyed by whole-body actions are processed. It would be particularly interesting to determine whether the relationships between the perception of emotions and/or traits like untrustworthiness conveyed by whole-body actions are similar to those conveyed by the face.

Other potential uses for these stimuli include investigations of the perception of static and dynamic emotional body language. There are considerable differences in the way that static and dynamic facial expressions are processed (Kilts, Egan, Gideon, Ely, & Hoffman, 2003; Pitcher, Dilks, Saxe, Triantafyllou, & Kanwisher, 2011). Importantly, processing of static images of whole-body actions and dynamic actions interacts (Barraclough & Jellema, 2011; Barraclough, Xiao, Oram, & Perrett, 2006). To our knowledge, research into the perception of emotional body language has only investigated the perception of static images and dynamic action separately. This database allows for individual frames to be taken from the videos in order to generate static images of actors conveying different traits. As video was filmed in HD, stills taken from this video are of high quality with many pixels (1,920 × 1,080). These stimuli can therefore be used to compare the perception of both static and dynamic emotional body actions across multiple actors, traits, and actions.

Our use of chroma key compositing techniques in generating these action videos allows for their flexible use. All actors are isolated from a uniform (currently, black) background. Many current methods of presenting videos during experiments (e.g., MATLAB, ePrime, Experiment Builder, etc.) allow users to set single colors within a video to be transparent. The black background of all the videos in the database can therefore be set to be transparent and actors superimposed on alternative backgrounds and environments. This allows for testing of the role of contextual visual information in the perception of traits from whole-body actions. Furthermore, by setting the background as transparent and shifting the position of the actors in the fronto-parallel plane, layers of simultaneous videos can be combined. This process would allow the generation of simple and controlled crowd scenes.

There are some limitations in the database that need to be mentioned. First, actions in the stimulus database were performed by both actors in training and nonprofessional actors. Previous research, however, has indicated that emotional actions produced by professional and nonprofessional actors are similar (Barliya, Omlor, Giese, Berthoz, & Flash, 2013; Roether et al., 2009). When incorporating actor type as a factor in our analysis, we found a small but significant difference that indicated that nonprofessional actors performed actions with slightly greater intensity than actors in training; this might indicate that nonactors are less able to portray subtle emotional signals, as compared to actors. Second, the action videos are currently available only in a limited video format. There are hundreds of available video formats and codecs, and these change regularly. We have decided to provide the 2-D videos in the Microsoft .wmv format (see the Method–Stimuli section), as this format can be widely used by various video playback engines. Three-dimensional versions of each video are available in .mp4 format. Third, participants only rated the 2-s versions of the videos of the actions. This was done to ensure that the rating experiment was carried out in a manageable time period.

In conclusion, we have generated and made publically available a large stimulus set of HD videos of whole-body human actions for use in psychological and neuroscientific research. Videos of multiple actors performing multiple actions while conveying different traits with different levels of intensity allow for flexibility in their combination to enable testing of various theoretically motivated hypotheses relating to the perception of human action and underlying processing mechanisms.

## Electronic supplementary material

Below is the link to the electronic supplementary material.

ESM 1 (PDF 295 kb)

ESM 2 (PDF 98 kb)

ESM 3 (PDF 76 kb)

ESM 4 (PDF 70 kb)

ESM 5 (PDF 69 kb)

ESM 6 (XLSX 14 kb)

## Footnotes

1 To examine whether actor type was important, we performed a three-way ANOVA with the factors of actor type (2) × trait (5) × stimulus trait intensity (4). Nondrama students conveyed the traits with significantly more intensity (*M* = 4.01, *SD* = 0.99), as compared with drama students (*M* = 3.86, *SD* = 0.91), indicating that nondrama students were just as capable of conveying emotions via their whole-body actions [main effect of actor type: *F*(1, 9) = 21.45, *p* = .001, *η*_p_^2^ = .70]. This difference was evident for all traits except anger, in which the intensity conveyed by the drama and the nondrama actors was equivalent [actor type × trait interaction: *F*(4, 36) = 4.19, *p* = .007, *η*_p_^2^ = .32]. There was no interaction between actor type and intensity, *F*(3, 27) = 2.71, *p* = .65, *η*_p_^2^ = .23, and a significant interaction between actor type, trait, and intensity, *F*(12, 108) = 3.40, *p* < .0001 *η*_p_^2^ = .27. All other terms had the same statistical significance reported.
