# Supplementary material for: A database of whole-body action videos for the study of action, emotion, and untrustworthiness
Source: Behav Res Methods. 2014 Mar 1;46(4):1042–51. doi: 10.3758/s13428-013-0439-6 (PMC4237924; doi:10.3758/s13428-013-0439-6)
Supplement: Supplementary file 1 — (PDF 295 kb) [file 13428_2013_439_MOESM1_ESM.pdf]

Action database readme

The database is organised across three folders:
Files for downloading
Files for viewing
Supplementary information

To <u>download</u> the files in the database enter *files for downloading*, click on the zipped file and then click on download.

To <u>view</u> the files in the database enter *files for viewing,* videos are organised into folders by the trait name. For example, all the angry actions can be found in the folder named 'angry'. When viewing files Google Drive typically requires a few minutes to the load the files, prior to this an error message may be received when trying to play a video.

The folder entitled, 'neutral rated for traits' contains the neutral actions rated along each of the five traits. These are contained within subfolders. For example, the 'neutral rated along the angry continuum' folder contains all the neutral actions labelled according to their average trait intensity rating on the angry continuum (see methods section of the paper for details).

The *supplementary information* folder contains a range of useful further information

actor details	- general information on the 29 actors
actor face photos	- full frontal photographs of each actor
number of stimuli by trait and action	- table detailing the number of stimuli by trait and action
number of stimuli by trait, action, and rating	-tables detailing the number of stimuli by trait, action, and rating
supplementary figures	- supplementary figures
trait identification stats summary	- summary statistics for the trait identification data
video error notes	- details of any potential anomalies in videos
video names readme	- explanation of how to interpret the naming convention for the videos
video rating data	- data from the ratings experiment and pivot table for stimulus selection
video identification data	- data from the trait identification and ratings experiments
video rating data readme	- explanation of how to use '*video rating data'*
walking video durations	- data on the durations of all walking videos
