# Supplementary material for: A database of whole-body action videos for the study of action, emotion, and untrustworthiness
Source: Behav Res Methods. 2014 Mar 1;46(4):1042–51. doi: 10.3758/s13428-013-0439-6 (PMC4237924; doi:10.3758/s13428-013-0439-6)
Supplement: Supplementary file 2 — (PDF 98 kb) [file 13428_2013_439_MOESM2_ESM.pdf]

Actor number	Filename	Age	Gender	Department	Studying
1	001M	38	M	Psychology	Lecturer
2	002F	20	F	Psychology	Psychology
3	003M	21	M	Theatre film and television	Writing, Directing and Performance
4	004F	20	F	Psychology	Psychology
5	005M	29	M	Psychology	Psychology Post Doc
6	006F	26	F	Psychology	Psychology PhD
7	007F	18	F	Theatre film and television	Writing, Directing and Performance
8	008M	20	M	Theatre film and television	Writing, Directing and Performance
9	009F	21	F	Theatre film and television	Writing, Directing and Performance
10	010F	21	F	Theatre film and television	Writing, Directing and Performance
11	011F	19	F	English	English Literature and Linguistics
12	012M	33	M	Psychology	Psychology
13	013F	19	F	Theatre film and television	Writing, Directing and Performance
14	014F	19	F	Theatre film and television	Writing, Directing and Performance
15	015M	22	M	Theatre film and television	Writing, Directing and Performance
16	016F	19	F	Psychology	Psychology
17	017F	20	F	Psychology	Psychology
18	018F	21	F	Politics	Politics
19	019M	19	M	Theatre film and television	Writing, Directing and Performance
20	020F	19	F	Theatre film and television	Acting
21	021M	32	M	Psychology	Psychology
22	022F	22	F	Psychology	Psychology
23	023F	21	F	Psychology	Psychology
24	024F	19	F	Maths	Maths

25	025M	28	M	Physical Education	Teacher
26	026F	24	F	Theatre film and television	Writing, Directing and Performance
27	027M	19	M	Theatre film and television	Writing, Directing and Performance
28	028F	23	F	Psychology	Psychology
29	029F	20	F	Psychology	Psychology

No. of Males	No. of Females	No. TFTV	No. Psychology	No. other dept
10	19	12	13	4
