# Supplementary material for: A database of whole-body action videos for the study of action, emotion, and untrustworthiness
Source: Behav Res Methods. 2014 Mar 1;46(4):1042–51. doi: 10.3758/s13428-013-0439-6 (PMC4237924; doi:10.3758/s13428-013-0439-6)
Supplement: Supplementary file 3 — (PDF 76 kb) [file 13428_2013_439_MOESM3_ESM.pdf]

Trait	Neutral	Angry	Fearful	Happy	Sad	Untrustworthy
No. of stimuli	174	522	522	522	522	521

Action	Jump	Put down box	Pick up box	Sit down	Stand and act	Walking
No. of stimuli	464	464	464	464	463	464

Not recorded due to a filming error: 027m_unt_2_sta
