# Supplementary material for: A database of whole-body action videos for the study of action, emotion, and untrustworthiness
Source: Behav Res Methods. 2014 Mar 1;46(4):1042–51. doi: 10.3758/s13428-013-0439-6 (PMC4237924; doi:10.3758/s13428-013-0439-6)
Supplement: Supplementary file 4 — (PDF 70 kb) [file 13428_2013_439_MOESM4_ESM.pdf]

Key for video file names

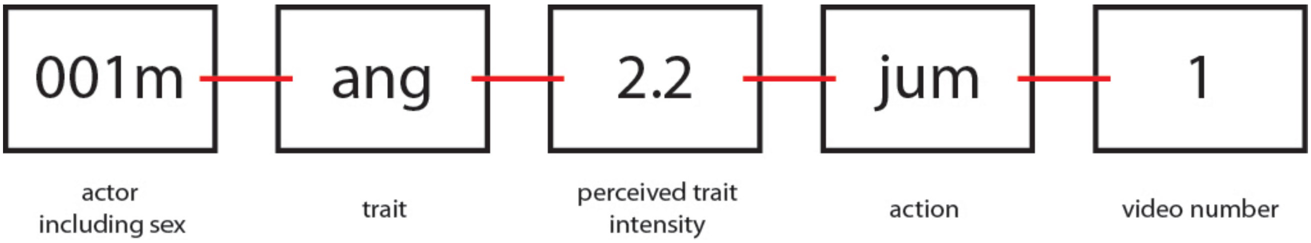

Actor
001 – 029
f = female
m = male

Trait
ang = angry
fea = fearful
hap = happy
neu = neutral
sad = sad
unt = untrustworthy

Perceived trait intensity
1 – 9

Action
sta = standing and acting
pdb = put down box
pub = pick up box
sit = sitting
jum = jumping
win = walking

Number
Incremental number for multiple instances of videos with identical actor, trait, perceived trait intensity and action
