# Supplementary material for: A database of whole-body action videos for the study of action, emotion, and untrustworthiness
Source: Behav Res Methods. 2014 Mar 1;46(4):1042–51. doi: 10.3758/s13428-013-0439-6 (PMC4237924; doi:10.3758/s13428-013-0439-6)
Supplement: Supplementary file 5 — (PDF 69 kb) [file 13428_2013_439_MOESM5_ESM.pdf]

Help to use video rating data.xls

Tabs:
raw data
Contains all data from the experiment where 10 observers rated all videos

The FileName column contains the original file naming system for the videos. Here, the number after the trait indicates the intensity at which the actor was asked to act that trait where 1=low, 2=medium, 3=high e.g. ang3 would be angry acted at a high intensity. For neutral actions, the number was always 0, giving neu0. This information becomes effectively redundant when using the new file naming convention that includes the average perceived trait intensity.

00 in the filename refers to camera from which the video was filmed (left camera of the 3D camera).

data pivot
Contains pivot table that references raw data. This pivot table can be used to select stimuli for experiments.
